# The role of retinal Müller cells in diabetic retinopathy and related therapeutic advances

**DOI:** 10.3389/fcell.2022.1047487

**Published:** 2022-12-02

**Authors:** Shuo Yang, Shounan Qi, Chenguang Wang

**Affiliations:** Department of Ophthalmology, The Second Hospital of Jilin University, Changchun, China

**Keywords:** diabetic retinopathy, retinal Müller cells, neurodegeneration, inflammation, treatment

## Abstract

Diabetic retinopathy (DR) is a significant complication of diabetes. During the pathogenesis of retinal microangiopathy and neuronopathy, activated retinal Müller cells (RMCs) undergo morphological and structural changes such as increased expression of glial fibrillary acidic protein, disturbance of potassium and water transport regulation, and onset of production of a large number of inflammatory and vascular growth factors as well as chemokines. Evidently, activated RMCs are necessary for the pathogenesis of DR; therefore, exploring the role of RMCs in DR may provide a new target for the treatment thereof. This article reviews the mechanism of RMCs involvement in DR and the progress in related treatments.

## 1 Introduction

**FIGURE 1:**
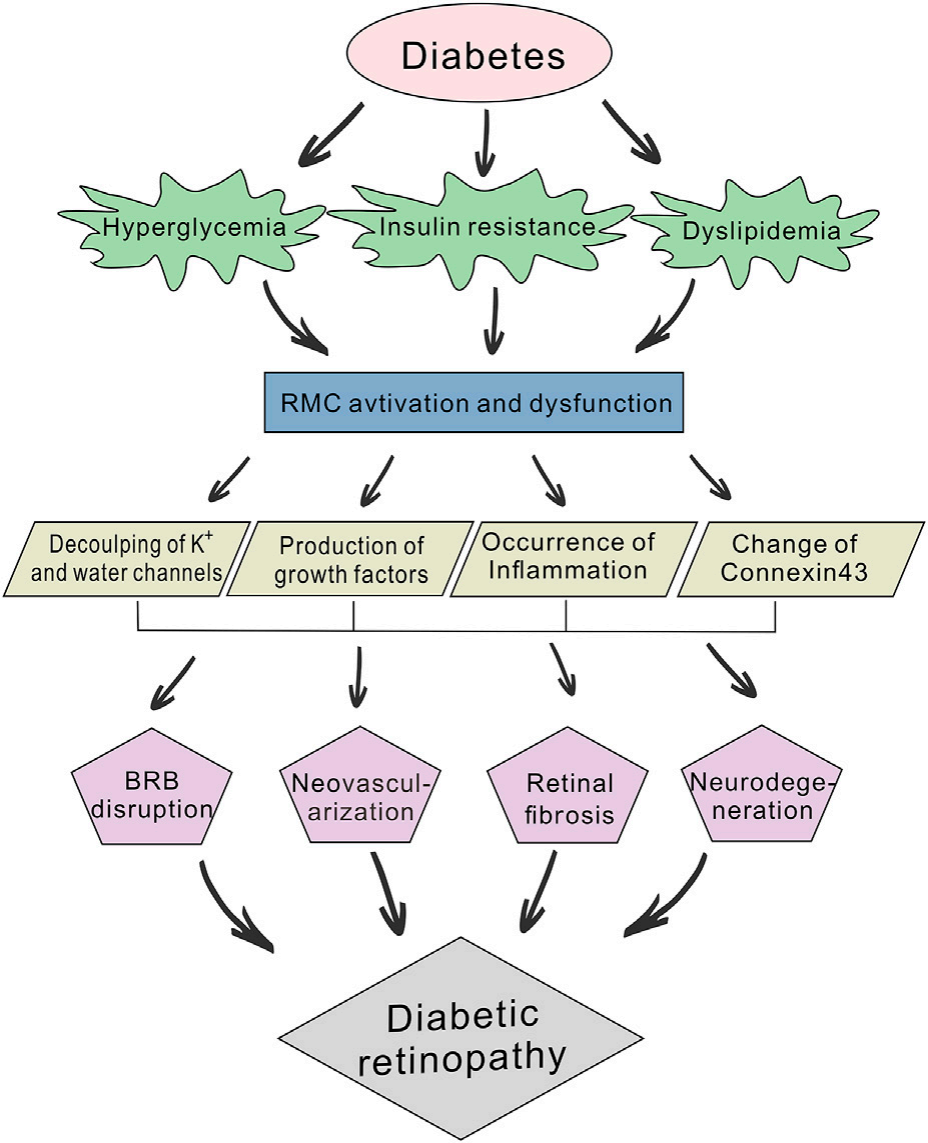
Schematic diagram of the pathogenic mechanisms of diabetic retinopathy (DR). Diabetes can lead to abnormal glucose and lipid metabolism, while hyperglycemia, insulin resistance and dyslipidemia will further accelerate the development of DR. Prolonged hyperglycemia induces the abnormal activation and dysfunction of retinal Müller cells (RMCs) and affects their physiological function. This involves the decoupling of potassium and water channels, production of growth factors (e.g., TGF-β, VEGF and FGF), occurrence of inflammation and changes in Connexin43 expression. RMC dysfunction also participates in blood-retinal barrier (BRB) disruption, neovascularization, retinal fibrosis, and neurodegeneration.

The retina is a classic “neuro-vascular coupling” tissue and coordinates the bioactivity of neurobioloy retinal blood flow. Diabetic retinopathy (DR) is characterized by retinal nerve deformation and microcirculatory disturbances, and its pathogenesis reflects the interactive nature of this “neuro-vascular coupling” (Moran et al., 2016; Sinclair et al., 2019). According to different needs derived from either its standard physiology or pathological processes, the retina regulates a variety of different cell types including vascular endothelial cells, retinal glial cells, retinal pigment epithelium cells, and photoreceptor cells; as well as inflammatory and growth factors (Kugler and Greenwood, 2021). The cascade reaction triggered by hyperglycemia as an initiating factor is not only concentrated in retinal microangiopathy, early neuronal apoptosis and glial cell activation are also participate in the destruction of blood-retinal barrier (BRB), water and ions transport disorder (Rao et al., 2019), release of growth factors (Gaonkar et al., 2020), and inflammation (Schmalen et al., 2021), all of which act as injury accelerating factors that promote the vicious circle of neurodegeneration and microangiopathy, resulting in irreversible retinal dysfunction (Figure 1).

Exploring the pathogenesis of DR from a cellular perspective has attracted increasing attention from researchers worldwide. Retinal Müller glial cells (RMCs), as the main macroglia in the retina, span the entire thickness of the tissue, and ensheath all retinal neurons and microvascular of retina (Wang et al., 2017). This morphological characteristic is reflected by a multitude of functional interactions between neurons and the BRB, which play fundamental roles in DR. In its early stages, neurotrophic factors secreted by activated RMCs can protect the retina by alleviating retinal edema and protecting optic ganglion cells (Ou et al., 2020). However, in late stages, the cytokines and inflammatory factors secreted by the activated RMCs damage the BRB, increasing apoptosis, and abnormal secretion of cytokines. This suggests that RMCs can be a potential target for DR treatment (Becker et al., 2021). Therefore, new strategies may be developed by further exploring the role and mechanism of RMCs in DR pathogenesis. In this paper, the role of RMCs in the development and progression of DR are reviewed.

## 2 Distribution and function of RMCs in the retina

The RMCs account for approximately 90% of retinal glial cells and run through the whole layer of the retina (Subirada et al., 2018). The cell bodies of RMCs are found in the inner nuclear layer (INL), whereas their ends wrap the neurons and blood vessels in the ganglion cell layer to form the internal limiting membrane. They then form tight junctions with photoreceptor cells to form the external membrane (Gao et al., 2021). RMCs are not only involved in maintaining the structural integrity of the retina but are also essential its homeostasis and physiological function. First, RMCs are interconnected to form a reticular scaffold in which neurons are wrapped, providing support to them. They can regulate neuronal excitability and protect neurons from excitotoxicity by taking up and recycling neurotransmitters such as GABA and glutamine (Singh et al., 2020). They also participate in retinal glucose metabolism and provide nutrition for the oxidative metabolism of retinal neurons (Goldman 2014). Second, RMCs are rich in ion channels, ligands, receptors, membrane-spanning transporters, and enzymes that help to maintain water and ions homeostasis in retinal tissues (Beverley and Pattnaik, 2022). In addition, RMCs play an important role in retinal innate immunity and inflammation through phagocytosis of cellular debris, release of inflammatory mediators, and regulation of immune cells such as microglia (Vecino et al., 2016).

## 3 RMCs and DR

**FIGURE 2:**
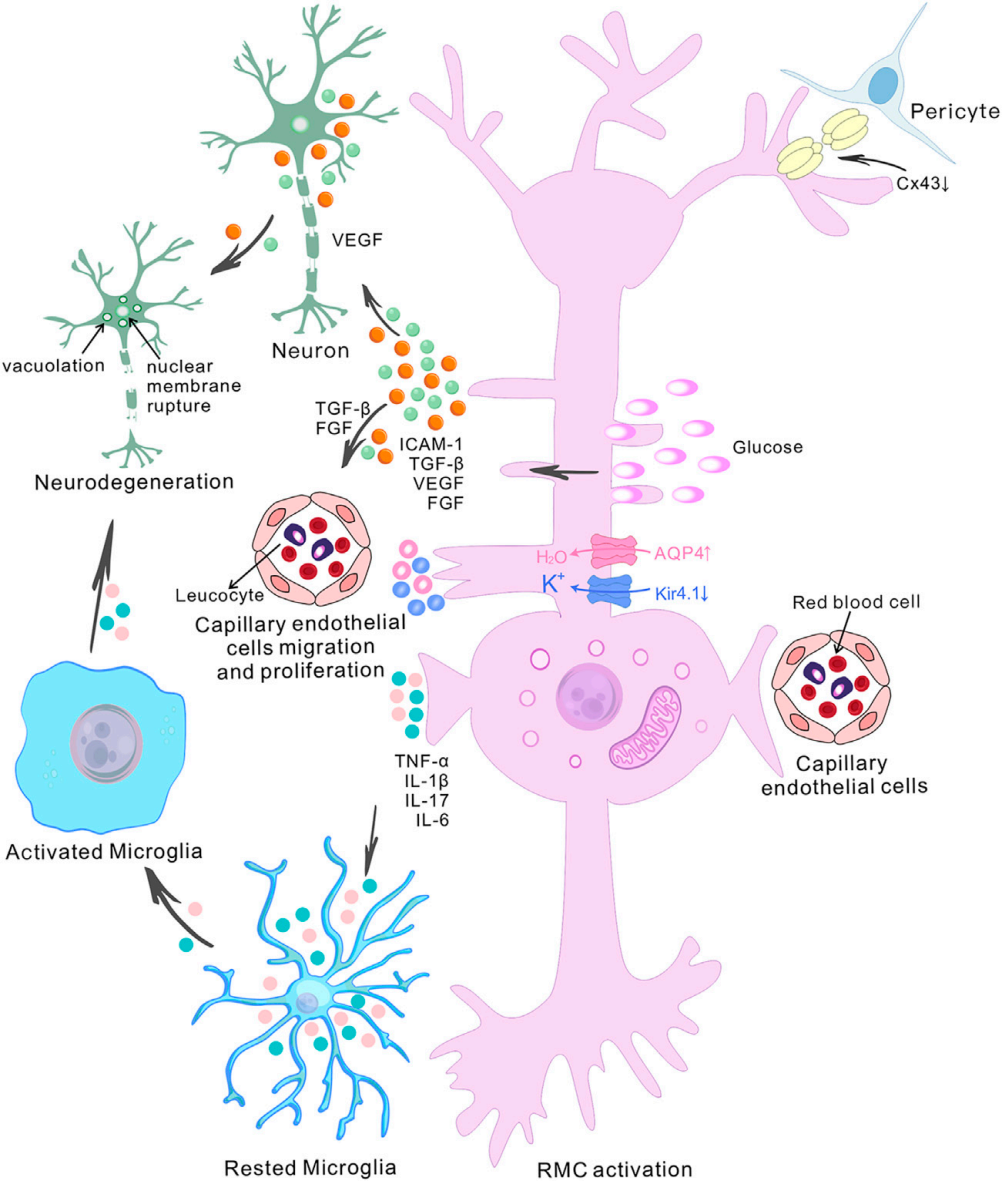
The role of retinal Müller cells in the pathogenesis of diabetic retinopathy. The diagram shows the central role of retinal Müller cells (RMCs) in various pathophysiological events in diabetic retinopathy (DR) and depicts the interactions between RMCs, capillary endothelial cells, pericytes and neurons. High glucose-induced RMC activation and Connexin43 downregulation in pericytes and Müller cells ultimately lead to accelerated retinal cell death. The accumulation of water and ions in RMCs is a pathogenic factor involved in retinal degeneration during DR. It has been found that there is upregulation of aquaporin 4 (AQP4) and down-regulation of the inwardly-rectifying potassium channel 4.1 (Kir4.1) in diabetes. Abnormally-activated RMCs produce pro-inflammatory mediators such as TNF-α, IL-1β, IL-17 and IL-6, and growth factor such as TGF-β, VEGF and FGF. The release of VEGF by RMCs exacerbates retinal neurodegeneration, and the release of TGF-β and FGF induces the migration and proliferation of capillary endothelial cells. Increased pro-inflammatory cytokines contribute to microglial activation. Activated microglial also produce increased levels of pro-inflammatory molecules, which then induce neuronal degeneration, altogether contributing to the development of DR.

RMCs are the main glial cells in the retina and are essential for maintaining retinal homeostasis. They not only penetrate the whole layer of the tissue, but also form the anatomical connection between the retinal neurons and retinal blood vessels, the vitreous body and the subretinal space. This morphological relationship is reflected in the multiple functions of RMCs (Bringmann et al., 2006). Thus, once the microenvironment is disturbed by factors such as chronic hyperglycemia, RMC becomes dysfunctional, which is manifested as water-potassium imbalance leading to cellular edema, the production of pro-angiogenic factors leading to neovascularization, the establishment of a chronic inflammatory environment, and the dysregulation of neuronal function, and subsequent the morphological structure changes (Figure 2).

### 3.1 Pathological changes of RMCs during the pathogenesis of DR

Confocal images of rat RMCs under high glucose conditions showed mitochondrial disruption and abnormal membrane potentials. Additionally, decreased cellular oxygen consumption rates and maximal oxygen consumption rates were measured by the bioenergetic assay (Tien et al., 2017). The results of electron microscopy and histochemical studies on the human retina by Fehér et al. (2018) showed that the basement membranes of RMCs were significantly thickened in the early stages of DR, with embedded translucent round vacuoles and highly dense granules, indicating the presence of lipids. As the severity of diabetes increased, RMCs gradually developed the neuroglial phenotype of poorly differentiated cell clusters. In addition, the number of mitochondria, lysosomes, endoplasmic reticula, and Golgi apparatus was reduced while that of vacuoles was increased. Additionally, it was found that the morphology of RMCs was significantly altered in the diabetic rat model, which exhibited deformed and denser nuclei, more dispersed nuclear chromatin, and increased cytoplasmic glycogen, dense bodies, and lysosomes. These morphological changes became more evident around the capillaries with the development of DR (Sorrentino et al., 2016). Studies have also found that as DR progresses, RMCs gradually start to undergo apoptosis, and the frequency of apoptosis is rapidly accelerated due to a decrease of protective cytokines such as BDNF and GDNF (Fu et al., 2015). These findings suggest that the altered RMCs ultrastructure and impaired organelles leading to cellular hypofunction may contribute to the development of DR.

Under normal conditions, glial fibrillary acidic protein (GFAP) is primarily expressed by retinal astrocytes, but not by RMCs (Li D. et al., 2020). One of the striking signs indicating that RMCs are activated during diabetic disease is the expression of GFAP, a characteristic molecular marker of RMCs injury and reactive gliosis (Sanchez et al., 2022). In animal models of diabetes, GFAP was found to be highly expressed in RMCs, while its expression was reduced or absent in astrocytes (Barber et al., 2000). It was also demonstrated that high GFAP expression is secondary to the activation of RMCs, which in turn leads to neuronal and microvascular damage (Picconi et al., 2019). It was found that the average concentration of GFAP in the aqueous humor was higher in DR patients compared to healthy controls or diabetic patients without DR. Additionally, optical coherence tomography showed that the inner nuclear layer was thinner in DR patients (Vujosevic et al., 2015).

### 3.2 Involvement of RMCs in the pathogenesis of DR

Changes in the homeostasis of the retinal microenvironment will disrupt the normal function and homeostasis of RMCs, affecting in turn the entire retina. As mentioned earlier, activated RMCs play an important role in various pathological changes in DR, inducing abnormal water and ion transport, neovascularization, secretion of inflammatory factors, and changes in retinal neurons.

#### 3.2.1 Abnormalities in potassium (K^+^) and water channels caused by active RMCs

Under normal circumstances, K^+^ produced by the excitation of nerve tissue and water produced by tissue metabolism are released into the interstitial space. The K^+^ influx into RMCs stimulates the inwardly-rectifying potassium channel 4.1 (Kir4.1), which actively discharges K^+^ into capillaries. Previous studies have shown that aquaporin 4 (AQP4) channels promote the movement of water through the glial-vascular interface and are related to the excretion of K^+^ ions from glial cells into the blood (Rao et al., 2019). Kir4.1 and AQP4 channels show a structural and functional coupling, jointly promoting the transmembrane transport of excessive water and K^+^ into the retinal interstitial space (Ruiz-Ederra et al., 2007). However, under the pathological conditions of high glucose, AQP4 expression is gradually increased and Kir4.1 expression is significantly decreased in the later stages of the disease. Consequently, the dynamic balance between the AQP4 and Kir4.1 is disrupted, causing an uncoupling of the channels and leading to abnormal water and K^+^ transport, resulting in swelling of RMCs and retinal vascular leakage (Vujosevic et al., 2015).

Kir4.1 is the main channel protein in RMCs that undertakes drainage functions. In the healthy retina, it is predominantly distributed in the RMCs terminals, the inner limiting membrane, and around the middle and deep capillary plexus. Kir4.1-mediated homeostasis of the retinal microenvironmental in RMCs is essential for the retina to maintain normal physiological functions, which not only mediates K^+^ homeostasis but is also coupled with water transport in RMCs. Together, they maintain osmotic homeostasis between the inside and outside of the cells, thus ensuring optimal functioning of RMCs (Gao et al., 2018). It has been shown that Kir4.1 channel activity is reduced in proliferative diabetic retinopathy (PDR) compared to non-proliferative diabetic retinopathy (NPDR), leading to RMCs depolarization, which impairs voltage-dependent functions such as uptake of glutamate, GABA, and other neurotransmitters (Alex et al., 2020). Studies have shown that in DR, the downregulation of Kir4.1 in RMCs leads to a decrease in cell drainage capacity, the swelling of RMCs, and a decrease of fluid removal in the macular. This causes fluid accumulation which can lead to macular thickening, destruction of the retinal structure, and impaired visual function (Hassan et al., 2017). After Kir4.1 downregulation in RMCs, the increase in extracellular K^+^ concentration depolarizes neurons and glial cells, leading to glutamate accumulation. The increase in glutamate concentration then leads to a decrease in glutamate-aspartate transporter (GLAST) expression, resulting in abnormal synaptic transmission. This, in turn, affects the function of action potential-generating neurons in the inner retina, ultimately leading to irreparable visual impairment (Gao et al., 2018).

AQP4 is a bidirectional transmembrane transporter protein that co-localizes and interacts with Kir4.1, mediating the reabsorption of water from the extracellular space of the retina back into the blood or transport thereof to the vitreous humor. The fluid in the blood or vitreous humor can also enter RMCs through AQP4 and then enter the extracellular space of the retina through RMCs (Schey et al., 2014). Under normal conditions, RMCs transport excess water from the retina to the blood through the cross-cell water transport channel through AQP4. In diabetes, the expression of AQP4 on RMCs increases significantly, and the fluid from the retinal microvasculature can thus enter the retinal cells in large quantities due to hydrostatic pressure; this excess fluid cannot be reabsorbed back into the blood in time, which eventually causes retinal edema (Kida et al., 2017). AQP4 expression was found to be significantly increased in the rat retina during diabetes, especially in RMCs (Amann et al., 2016). AQP4 levels in the aqueous humor were also significantly elevated in patients with diabetes (including those without clinical features of retinopathy). In a group of patients affected by DR, AQP4 levels were approximately 25 times higher than in healthy participants and four times higher than in diabetic patients without DR. This shows that the expression of AQP4 is markedly increased in both DR and preclinical DR patients. Therefore, AQP4 is considered an early marker of RMCs alterations in diabetes (Vujosevic et al., 2015).

RMCs are essential for fluid transport in the retina, and AQP4 and Kir4.1 play a key role in maintaining the retinal “dry state”. Under high glucose conditions, RMCs fail to properly regulate the transport of ions and water in the retina, which is one of the important mechanisms underlying the occurrence and development of DR. It has been suggested that DR can be prevented and treated by targeting the liquid transport pathway in RMCs.

#### 3.2.2 Abnormal expression of Connexin43 in RMCs

A gap junction is an area of contact between adjacent cells that allows small molecules to be transported directly between cells. A gap junction channel is composed of two hemichannels, each of which is in turn composed of six connexins. Connexin43 (Cx43) is the most widely expressed connexin, and it is abundantly present on the apical protrusions of RMCs, which are involved in maintaining the vascular permeability of the retina and the structure of the BRB, as well as in regulating cell apoptosis (Danesh-Meyer et al., 2016). It was observed that the expression of Cx43 was decreased in RMCs under high glucose conditions, which subsequently inhibited gap junction intercellular communication (GJIC) and led to protein kinase B inactivation, ultimately leading to RMC apoptosis. Furthermore, high glucose levels induced the downregulation of Cx43 and impairment of GJIC function in RMCs co-cultured with normal pericytes, eventually resulting in apoptosis of RMCs and pericytes. This suggests that Cx43 plays an important role in intercellular communication between these cells (Muto et al., 2014). GTPases play a vital role in regulating intracellular transport and facilitating the translocation of fusion proteins to the cell surface. The GTPase named Rab20 was shown to be involved in regulating Cx43 translocation. It was found that Rab20 expression was significantly elevated in RMCs under high glucose conditions, which hindered the localization of Cx43 to the cell surface. As a consequence, intercellular communication between retinal endothelial cells and RMCs becomes impaired, thus promoting the loss of DR-related retinal vascular endothelial cells and RMCs. However, the use of Rab20 siRNA inhibited Rab20 overexpression and improved GJIC function, thus preventing high glucose-induced neurovascular injury (Kim et al., 2020). Therefore, the abnormal downregulation of Cx43 in RMCs induced by high glucose may promote the disruption of neurovascular homeostasis associated with DR.

#### 3.2.3 RMCs and growth factors

Hyperglycemia promotes the secretion of growth factors, such as vascular endothelial growth factor (VEGF) (Rezzola et al., 2021), fibroblast growth factor (FGF) (Li et al., 2019), insulin-like growth factor-1 (IGF-1) (Actis Dato et al., 2021), and transforming growth factor-β (TGF-β) (Schmalen et al., 2021), which are involved in the regulation of neovascularization and cause retinal fibrosis in DR. It has been found that VEGF expression in RMCs in the high-glucose state increases with the duration of disease, which in turn increases capillary permeability, promotes neovascularization, and ultimately leads to irreversible visual impairment (Capozzi et al., 2016). The role of VEGF secreted by RMCs in DR was first investigated by Y.Z.Le et al., who used an inducible Cre/*lox* system to create conditional knockout mice. VEGF levels in this conditional VEGF-knockout model were reduced by 47.4% in RMCs compared to those in wild-type mice, and the typical pathological changes in DR such as reduced leukocyte adhesion, capillary non-perfusion, vascular leakage, and neovascularization were also ameliorated (Wang et al., 2010). The number of ferritin particles and the VEGF content were increased in RMCs from knockout mice for TIM-2, a ferritin-specific binding receptor found mainly in this cell type. This resulted in impaired paracellular and transendothelial transport, and in eventual disruption of the BRB, suggesting that iron overload caused by TIM-2 knockout may be a new mechanism for VEGF involvement in DR (Valença et al., 2021).

Fibroblast growth factor (FGF) is a neurotrophin and mitogen that not only mediates neuronal differentiation, survival, and regeneration; but also acts directly on vascular endothelial cells, influencing neovascularization (Xie et al., 2020). The expression of FGF was found to be significantly increased in RMCs in high glucose environments, and was directly proportional to the increase in glucose concentration in the culture medium. This then induced the expression of various pro-vascular growth factors and pro-inflammatory cytokines, such as VEGF, interleukin IL-1β, IL-6, and interferon-γ (Rezzola et al., 2021).

Insulin-like growth factor-1 (IGF-1) is a trophic factor in the retina that is a crucial in both normal and pathological conditions (e.g., the formation of retinal neovascularization in DR). It increases glucose transport to the retinal vascular endothelium of the retina through glucose transporter 1 (GLUT1) expressed in RMCs, and stimulates RMCs activation, increasing their mobilization to the retinal stroma and thus causing retinal detachment in PDR (Xi and Wai, 2019; Actis Dato et al., 2021).

The interaction between TGF-β receptors and their ligands leads to overexpression of extracellular matrix proteins, which results in retinal fibrosis, and is involved in the proliferation of fibroblasts and vascular endothelial cells (Peng et al., 2022). It was found that significantly activated RMCs in patients with PDR secreted large amounts of TGF-β and promoted their own transdifferentiation into myofibroblasts through glial–mesenchymal transition, which is involved in diabetic fibrovascular proliferation (Wu et al., 2020).

The characteristic pathological change of DR is neovascularization. The above-mentioned studies confirmed that growth factors such as VEGF, FGF, IGF-1, and TGF- β are involved in the pathophysiology of DR. Although numerous factors are closely associated with DR. In terms of clinical application and overall perspective, there is often the synergistic effect of multiple factors in the progression of the disease. Therefore, more attention should be paid to the interaction of different growth factors in DR.

#### 3.2.4 RMCs and inflammation

Chronic inflammation of the retina is a key process in DR, which appears in the early stages of DR. Numerous studies have shown pathological changes in the retina of patients with DR due to chronic inflammation, including elevated inflammatory cytokines and chemokines, neutrophil and macrophage infiltration, vascular endothelial adhesion (leukocyte stasis), and disruption of the BRB (Forrester and Kuffova, 2020). *In vitro* experiments revealed that receptors for advanced glycation end products were overexpressed in RMCs exposed to high glucose levels. When bound to their ligands, these receptors could activate the mitogen-activated protein kinase (MAPK) signaling pathway, thereby upregulating the expression of pro-inflammatory cytokines (Rübsam et al., 2018). The gene expression profile of RMCs from streptozotocin (STZ)-induced diabetic rats showed that more than 70 genes were upregulated, and one-third of those were genes responsible for coding proteins related to the inflammatory response, such as IL-1β, tumor necrosis factor-α (TNF-α), intercellular cell adhesion molecule-1, vascular cell adhesion molecule-1, and integrin β-2; suggesting an important role of these inflammatory factors in DR (Gerhardinger et al., 2005).

In high glucose environments, the RMCs not only secrete IL-1β (a key regulator of DR inflammation), but also produce other inflammatory cytokines such as TNF-α and IL-6 (Schmalen et al., 2021). Additionally, the level of IL-6 was positively correlated with retinal macular thickness (Wang et al., 2020). Furthermore, IL-1β stimulates RMCs to secrete higher amounts of IL-8, an important activator and recruiter of leukocytes, by activating p38 MAPK and extracellular regulated protein kinase (ERK)-1/2,thus amplifying diabetic retinal inflammation (Liu et al., 2014). Another pro-inflammatory cytokine, IL-17A, affects cell function and survival through a dimeric receptor complex composed of IL-17RA and IL-17RC (Qiu and Liu, 2017). The IL-17RA-related Act1-TRAF6-IKK-NFκB signaling pathway was found to regulate IL-17A damage to RMCs in DR. The expression of IL-17RA in rat RMCs increased under high glucose conditions, and the use of anti-IL-17RA antibodies alleviated high glucose-induced RMCs dysfunction (Qiu et al., 2016).

Under the stimulation of inflammation, the expression of cell adhesion molecules and chemokines such as intercellular cell adhesion molecule-1 (ICAM-1), vascular cell adhesion molecule-1 (VCAM-1), and monocyte chemoattractant protein-1 (MCP-1) are also upregulated in RMCs, thus promoting the migration and adhesion of leukocytes to the retinal vessel wall, ultimately disrupting vascular permeability (Taghavi et al., 2019). Another driver of DR inflammation is CD40 expressed in RMCs, which triggers extracellular ATP release in RMCs and induces purinergic receptor (P2X7)-dependent secretion of pro-inflammatory factors by myeloid cells (Portillo et al., 2022). CD40 is a member of the TNF receptor superfamily, and CD40^−^CD40L pathway is the core of autoimmunity and adaptive immunity. The expression of CD40 gene and protein in retina of diabetic mice was upregulated (Portillo et al., 2016). Elevated levels of soluble CD40 receptor sCD40L in peripheral blood of patients with diabetes patients (Neubauer et al., 2010). Moreover, activation of CD40 upregulates the expression of ICAM-1 and MCP-1 in RMCs of diabetic mice, which enhances recruitment of leukocytes to the retina and contributes to neurovascular degeneration. Blocking CD40 signaling significantly inhibits the upregulation of these factors and attenuates retinal inflammation (Portillo et al., 2017). It was found that the expression of adhesion molecules (ICAM and VCAM) and chemokines (CCL2 and CXCL16) was significantly increased in RMCs treated with the pro-inflammatory cytokine TNF-α, and that CXCL6 was not only involved in leukocyte recruitment and inflammatory processes in DR but was also targeted to induce upregulation of nuclear factor kappa-B (NF-κB), phosphorylated ERK1/2, and VEGF synthesis and secretion in RMCs, thereby mediating the progression of inflammation and neovascularization in DR (Abu El-Asrar et al., 2020).

It can therefore be concluded that the inflammatory response mediated by RMCs plays an important role in the pathogenesis of DR. In response to prolonged hyperglycemia, RMCs secrete large amounts of inflammatory cytokines and form new vascular structures, while neovascularization in turn can result in further recruitment of RMCs and further release inflammatory cytokines. Persistent chronic inflammation eventually results in retinal vascular injury, tissue remodeling and loss of function.

#### 3.2.5 Effect of RMCs activation on retinal neurons

RMCs are important glial cells that maintain a close contact with each neurons and protect them by secreting neurotrophin, taking up and degrading glutamate, and secreting glutathione. An imbalance in the expression of neuroprotective factors is the main reason leading to retinal neuronal degeneration in the retina during diabetes. It was found that the concentrations of brain-derived neurotrophin and nerve growth factor, which are mainly synthesized and secreted by RMCs, were significantly decreased in the retinas of patients with diabetes, suggesting that RMCs play a fundamental role in the survival of retinal neurons (Fu et al., 2015). GLASTs in RMCs can remove excess glutamate from the synaptic gap, thus protecting neurons from glutamate excitotoxicity. GLAST activity in RMCs is reduced in a high glucose environment, which prevents the transfer of extracellular glutamate into RMCs, leading to an accumulation of glutamate in the retina and vitreous humor, causing cytotoxicity and eventually inducing irreversible death of retinal ganglion cells (RGCs). Therefore, glutamate toxicity damage to neurons caused by RMCs dysfunction is an important mechanism leading to DR (Wang et al., 2013).

It has also been proposed that activated RMCs do not always lead to the death of retinal neurons in DR, but may instead have neuroprotective effects against neurotoxicity induced by high glucose through the regulation of ERK1/2 phosphorylation (Matteucci et al., 2014). It has been found that RMC gliosis occurs in the early stages of DR, and these RMCs fill the spaces of neuronal necrosis, maintain the survival of residual neuronal cells, and regulate the stability of the neuronal internal environment, exerting neuroprotective effects. However, with the progression of DR, excessive gliosis can lead to retinal glial scar proliferation, affecting the regeneration and functional recovery of retinal neuronal cells, hindering the repair of retinal tissue structure, leading to RGC loss and decreasing the thickness of the retinal nerve fiber layer (Simó and Hernández, 2014). VEGF released from RMCs under high glucose conditions supports the survival of endothelial cells and retinal neurons in the early stages of injury and limits glucose-induced retinal vascular damage (de Hoz et al., 2016). Hyperglycemia increased IL-6 levels in human RMCs and the activation of IL-6 in the early stages induced the production of VEGF-A to protect RMCs from high glucose toxicity. However, as VEGF-A accumulates, it triggers angiogenesis and vascular breakdown or leakage, which accelerates the progression of DR (Coughlin and Trombley, 2019). Diabetic retinopathy can induce the release of nitrogen monoxide (NO) by RMCs. In the early stages, low concentrations of NO increase local retinal blood flow by dilating blood vessels, preventing platelet aggregation, and protecting neurons from glutamate toxicity by blocking N-methyl-D-aspartate receptor channels (Kobat and Turgut, 2020).

The neuroprotective effect of RMCs and some cytokines during the development of DR deserves attention by researchers. During the development of DR, this neuroprotective effect may be taken advantage of in the future by interfering with the morphological and structural changes, neurotransmitters secretion and signal transduction of RMCs.

## 4 Role of RMCs as a target of DR treatment

When RMCs are activated in diabetes, a variety of cytokines that mediate pathological changes in DR such as vascular leakage, pathological angiogenesis, and inflammation, are secreted. Therefore, therapeutic strategies that target RMCs will effectively prevent the loss of vision and aid in the early treatment of patients with DR (Rolev and Shu, 2021). Table 1 summarizes the different RMC targets that could potentially be used in the treatment of DR.

**TABLE 1:** Retinal Müller cells (RMCs) as a target for diabetic retinopathy (DR) treatment. The table shows current and novel treatment strategies in DR related to RMCs.

Drug	Target	Administration	Status for DR treatment	Results	Refenrence
2-HDP	Kir4.1	Oral	Preclinical	Protects against RMC, oxidative stress and K+ channel dysregulation	McDowell et al. (2018)
Metformin	Kir4.1	Oral	FDA approved	Corrects abnormal circadian rhythm and Kir4.1 channels	Alex et al. (2020)
Pinacidil	KATP channel	Intravitreous	FDA approved	Inhibits RMC gliosis and augments the expression of Kir4.1 in RMC	Li et al. (2021)
TGN-020	AQP4	Intravitreous	Preclinical	Suppress retinal edema in diabetic retinas	Oosuka et al. (2020)
Ranibizumab	VEGF	Intravitreous	FDA approved	Reduce endothelial cell proliferation, vascular leakage and neovascularization	Gross et al. (2018)
Bevacizumab	VEGF	Intravitreous	Off-label use	Similar clinical efficacy to ranibizumab	Glassman et al. (2020)
Conbercept	VEGF	Intravitreous	Phase 3 trial	Similar clinical efficacy to ranibizumab	Xia and Liu, (2021)
GLP1	VEGF	Eye-Drops	FDA approved	Arrests the progression of neurodegeneration and vascular leakage	Sampedro et al. (2019)
GSK2256294	IL-1β	—	Preclinical	Reduced palmitic acid- and IL-1β-stimulated RMC cytokine expression	Ontko et al. (2021)
SOCS1	Inflammatory factors	Eye drops	Preclinical	Prevent retinal neuroinflammation and early microvascular impairment	Hernández et al. (2019)
C-DIM12	NLRP3 inflammasome	Intragastric	Preclinical	Anti-inflammatory and neuroprotective	Li W. et al. (2020)
INCA-6	Inflammatory factors	intraocular	Preclinical	Inhibit RMC amplification of diabetic inflammation and endothelial pathogenic responses	Giblin et al. (2021)

### 4.1 Treatments targeting Kir4.1 and AQP4

In DR, the expression of Kir4.1 is reduced or is even absent in the perivascular and inner limiting membrane, leading to swelling of the RMCs. It was observed that the acrolein derivative FDP-lysine, an oxidative stress lipid oxidation end product, accumulated in RMCs of diabetic mice and downregulated Kir4.1 levels, while the acrolein scavenger 2-hydrazino-4,6-dimethylpyrimidine (2-HDP) prevented FDP-lysine accumulation and increased Kir4.1 expression in diabetic RMCs, and reversed diabetes-induced Kir channel deficiency in the perivascular region and RGC layer (McDowell et al., 2018). Other studies have found that metformin also improved the distribution and protein expression of Kir4.1 in RMCs from diabetic mice, as well as that of adenosine monophosphate (AMP)-activated protein kinase (AMPK), which is involved in lipid-lowering and insulin-sensitizing processes and interacts with Kir4.1 to maintain RMC osmotic homeostasis. Metformin improved RMCs dysfunction by activating AMPK in the treatment of type 2 diabetes (Alex et al., 2020). Pinacidil is a non-selective ATP-sensitive potassium channel opener. Intravitreal injection of pinacidil in diabetic rats significantly inhibited RMC activation and reversed diabetes-mediated Kir4.1 downregulation and AQP4 upregulation in RMCs (H. Li et al., 2021). The results showed that increased AQP4 expression significantly increased the volume of rat RMCs in a high-glucose culture medium and increased retinal thickness. The application of the AQP4 inhibitor 2-(nicotinamide)-1,3,4-thiadiazole (TGN-020), which inhibited AQP4 expression by intravitreal injection into rats, was found to inhibit the GFAP expression and reduce cell swelling of RMCs, thereby improving the extent of diabetic retinal edema (Oosuka et al., 2020).

### 4.2 Anti-VEGF therapy

RMCs are involved in VEGF production and in the induction of neovascularization in DR; therefore, VEGF inhibition with the aim of reducing vascular permeability and retinal neovascularization has become an important strategy to treat DR. Anti-VEGF drugs, including monoclonal antibodies (bevacizumab, ranibizumab, and ramucirumab) and recombinant fusion proteins (aflibercept and conbercept) have been widely used in the clinical setting, and new small-molecule inhibitors of VEGF receptors such as sorafenib, axitinib, apatinib, levatinib, and fruquintinib, have gradually entered the clinical trial stage (Chatziralli and Loewenstein, 2021). It was found that VEGF and AQP4 mRNA expression were increased and positively correlated in the RMCs of diabetic rats. Treatment of diabetic rat RMCs with bevacizumab revealed a significant decrease in VEGF and AQP4 mRNA expression, which reduced intracellular osmotic pressure in RMCs and reduced intracellular edema in RMCs (Wang et al., 2021).

Glucagon-like peptide-1 (GLP-1), is a peptide hormone that maintains glucose homeostasis by promoting insulin secretion and is widely expressed in the retina, plays a neuroprotective role in the central and peripheral nervous systems (Hernández et al., 2019). The b-wave amplitudes of ERG were significantly higher in diabetic mice treated with eye drops containing a GLP-1 agonist compared with the untreated group. Moreover, the treatment inhibited RMC activation downregulated VEGF mRNA levels, as well as the expression of genes coding for pro-inflammatory cytokines, such as IL-1β and IL-6. Additionally, GLP-1 agonist treatment can prevent the thinning of the neural retina caused by diabetes and restore the number of retinal cells to the same levels as in non-diabetic mice. The authors inferred that the topical application of a GLP-1 agonist blocked the progression of diabetic retinal neurodegeneration by downregulating VEGF and inflammatory cytokines and upregulating anti-apoptotic proteins, such as Bcl-xL and Akt (Sampedro et al., 2019).

VEGF is one of the most important factors that promote neovascularisation and increase vascular permeability. In the presence of hyperglycemia, RMCs are the main source of VEGF. Therefore, glial cell proliferation, neovascularization and vascular permeability can be reduced by regulating the VEGF signaling pathway in RMCs.

### 4.3 Anti-inflammatory therapy

Inflammation is involved in all stages of the disease, from NPRD to PDR, and also in diabetic macular edema (Buyuktepe et al., 2021). RMCs participate in the pathological process of inflammation through the secretion of various inflammatory mediators, such as IL-1β, TNF-α, and NF-κB. Therefore, anti-inflammatory therapy targeting RMCs can effectively alleviate ocular inflammation, thereby treating DR. Intraocular free fatty acid levels are significantly elevated in DR, and fatty acids can stimulate the expression of multiple DR-related signaling pathways in RMCs, including the NF-κB and the MAPK signaling pathways, inflammation, lipid signaling, and angiogenesis, resulting in leukocyte stasis, increased vascular permeability, and thickening of the vascular basement membrane (Eynard and Repossi, 2019). While cytochrome P450 cyclooxygenase exhibits anti-inflammatory activity by metabolizing fatty acids into epoxides, soluble epoxide hydrolase (sEH) inhibits its anti-inflammatory activity by reducing the half-life of epoxide. The use of the sEH inhibitor GSK2256294 in human-derived cell lines of RMCs in a high-glucose environment significantly downregulated the expression of fatty acids and IL-1β-induced inflammatory cytokines such as TNF-α and IL-8 in RMCs by blocking the NF-κB pathway. It has been suggested that this strategy could have a protective role by targeting RMCs inflammation in DR (Ontko et al., 2021). Suppressor of cytokine signaling 1 (SOCS1) is a negative regulator of cytokine signaling pathways in the SOCS family that ameliorates inflammation by inhibiting Janus kinase and regulating IFN-γ to attenuate the expression and transduction of cytokine signals such as IL-2, IL-6, IFN, TNF-α, and colony-stimulating factor. After 2 weeks of administration of SOCS1-derived peptide eye drops in diabetic mice, it was found that RMC activation was significantly inhibited, ERG parameters were significantly improved, and IL-1β, IL-6, TNF-α, and VEGF expression were downregulated, suggesting that local application of SOCS1-derived peptide could be used in the early treatment of DR by effectively blocking RMC inflammation (Hernández et al., 2019). TNF is a key regulatory component of the immune system and contributes to the initiation and maintenance of inflammation (Dostert et al., 2019). At present, there are five kinds of anti-TNF drugs in clinical use: infliximab, adalimumab, certolizumab pegol, golimumab, and etanercept (Monaco et al., 2015). Recently studies have showed that the presence of CD40 restricted to RMCs is sufficient to cause upregulation of inflammatory molecules such as TNF, IL-1β and ICAM-1 in the retina of diabetic mice and lead to the development of diabetic retinopathy (Portillo et al., 2017). Meanwhile, TNF receptor-associated factors (TRAFs) are critical regulators of CD40 (Bishop and Hostager, 2002). Portillo et al. (2022) have pointed out that CD40-TRAF (mainly involving TRAF2 and TRAF3) signaling in RMCs inhibits retinal inflammation and the development of DR. In addition, this study demonstrated that intravitreal administration of a cell-permeable CD40-TRAF2,3 blocking peptide impairs upregulation of TNF-α and IL-1β in diabetic mice. This approach offers therefore potential new targets to avoid inflammatory diseases such as DR. Although anti-TNF drugs have been widely used in the treatment of inflammatory diseases, the mechanism of their action in DR requires further study, especially how to target TNF or TNF receptors in RMC for earlier treatment of DR.

The nuclear receptor-related factor 1 (Nurr1) could suppress the inflammatory response by binding to NF-κB (the promoter of inflammation) and downregulating the downstream NOD-like receptor protein 3 (NLRP3) inflammasome. It was found that Nurr1 expression was downregulated in RMCs under high glucose condition, but NLRP3 inflammasome expression was upregulated. C-DIM12, a Nurr1 agonist, inhibits the activation of RMCs and loss of RGCs in STZ-induced diabetic mice, indicating that Nurr1 has anti-inflammatory and neuroprotective effects in DR (Li W. et al., 2020). Nuclear factor of activated T cells (NFAT) is a transcriptional regulator of inflammatory cytokines that is widely expressed in retinal cells and is present in the cytoplasm in a phosphorylated state under normal conditions and dephosphorylates upon inflammatory stimulation. NFAT shuttles to the nucleus to increase the transcription of inflammatory cytokines. It has been found that NFAT is involved in the downstream inflammatory signal transduction mediated by IL-1β in RMCs. The use of NFAT inhibitor (INCA-6) could downregulate the expression of IL-1β and TNF-α in experimental diabetes, suggesting that inhibition of NFAT attenuates diabetic-induced retinal inflammation (Giblin et al., 2021). In view of the anti-inflammatory properties of glucocorticoids (GCs) and the fact that glucocorticoid receptors are found almost exclusively in RMCs, application of corticosteroid agonists has been proposed as a therapeutic strategy against DR (Gallina and Zelinka, 2014). Glucocorticoid-induced leucine zipper (GILZ) is an important anti-inflammatory mediator of GCs and has great potential in anti-inflammatory therapy (Ronchetti and Migliorati, 2015). A study using an animal model of inflammatory disease showed that GLIZ exerts its anti-inflammatory activity by interacting with and suppressing the NF-κB and activator protein-1 (AP-1), et al. Moreover, GLIZ significantly downregulated the expression of inflammatory cytokines such as TNF-α, ICAM-1, and IL-1β in RMCs (Gu et al., 2018). Considered together, these results suggest that GLIZ could inhibit inflammatory responses in RMCs and may play an important role in the treatment of DR.

As we gain a more in-depth understanding of the inflammatory mechanism underlying DR, therapeutic strategies involving targets associated with inflammation continue to emerge. The therapeutic effect of drugs has been continuously improved, from single target inhibition to multi-target synergism, and from laboratory to clinic. The complex mechanisms by which multiple factors secreted by RMC interact with each other currently make the development of therapeutic strategies more difficult, so new advances in the study of the pathogenesis of inflammation in the development of DR will play a huge role in the precise treatment of DR.

## 5 Summary and future perspectives

In summary, in the pathogenesis of DR, prolonged hyperglycemia affects the normal physiological function of RMCs, leading to the disturbances in the retinal water-ion balance, disruption of the BRB, imbalance in the homeostasis of the retinal microenvironment, and the secretion of pro-inflammatory cytokines and chemokines following RMCs overactivation. This leads to inflammation and retinal glial scar proliferation, resulting in neuronal degeneration. It follows that in DR with retinal neurodegenerative lesions and microcirculatory dysregulation, alterations in RMCs trigger a cascade reaction, making them an important regulatory factor that cannot be ignored. However, the majority of the current research is still focused on the treating outcomes after the damage has already occurred, that is, how to inhibit neovascularization, how to prevent vascular leakage, and how to improve the success rate of vitreous surgery; whereas the triggering factors and initial changes responsible for causing the lesions have been mostly ignored.

We postulate that the activation of RMCs has an important effect on retinal nerve injury and vasculopathy in DR, and therefore it would be a new and potentially effective approach to treat DR and other related retinal diseases by interfering with RMC signaling and the inflammatory microenvironment, thus inhibiting, delaying, or even reversing the dysfunction of damaged RMCs. For example, RMCs are found throughout the entire retina and can therefore be a useful carrier for somatic gene therapy, providing nutrients to the retina and preventing or even reversing damage of optic nerve. However, there are still a number of issues that remain to be addressed. For example, the advantages and disadvantages of early activation of RMCs in DR, and how to reverse its overreaction to pathological stimulus. RMCs, as potentially differentiated progenitor cells in the retina, whether they could be a key target cell for DR repair, whether it signaling pathway could be used as an intervention target in DR treatment, and whether this can protect the optic nerve, inhibit neovascularization, vascular leakage and retinal inflammation at the same time is still not clear. Therefore, we need more evidence to understand the role that RMCs pluripotency plays in disease. Moreover, it is necessary to use the existing methods to explore advanced treatment strategies and pay attention to the basic research of RMCs in the context of DR.
